# Aspects of Medication and Patient participation—an Easy guideLine (AMPEL). A conversation guide increases patients’ and physicians’ satisfaction with prescription talks

**DOI:** 10.1007/s00210-021-02107-0

**Published:** 2021-06-09

**Authors:** Verena Kirsch, Jan Matthes

**Affiliations:** Centre of Pharmacology, University of Cologne, Gleueler Strasse 24, 50931 Cologne (Köln), Germany

**Keywords:** Health communication, Drug prescribing, Drug information, Guide, Shared decision-making

## Abstract

Patients want more information and active participation in medical decisions. Information and active participation correlate with increased adherence. A conversation guide, combining patient-relevant drug information with steps of shared decision-making, was developed to support physicians in effective and efficient prescription talks. Six GP trainees in community-based primary care practices participated in a controlled pilot study in sequential pre-post design. Initially, they conducted 41 prescription talks as usual, i.e., without knowing the guide. Then, they conducted 23 talks considering the guide (post-intervention phase). Immediately after the respective talk, patients filled in a questionnaire on satisfaction with the information on medication and physician–patient interaction, and physicians about their satisfaction with the talk and the application of the guide. Patients felt better informed after guide-based prescription talks (e.g., SIMS-D in median 10 vs. 17, p < 0.05), more actively involved (KPF-A for patient activation 2.9 ± 0.8 vs. 3.6 ± 0.8, p < 0.05), and more satisfied with the physician–patient interaction. Physicians rated the guide helpful and feasible. Their satisfaction with the conversation was significantly enhanced during the post-intervention phase. The evaluation of the duration of the talk was not influenced. Enhanced patients’ and physicians’ satisfaction with prescription talks encourages further examinations of the conversation guide. We invite physicians to try our guide in everyday medical practice.

**Supplementary Information:**

The online version contains supplementary material available at 10.1007/s00210-021-02107-0.

## Introduction

In most consultations, physicians prescribe a medication to their patients (Stevenson et al. 2000). It has been suggested that one of the most common and important decisions a patient can participate in is whether and how to use medicines (Makoul et al. 1995). For patients, physicians are one of the most important sources of drug information (Nink and Schröder 2005; Tarn et al. 2009b). In fact, patients want more information about their drug therapy, and often more than their physicians assume (Nair et al. 2002; Twigg et al. 2016). It has been shown that better information about a treatment is associated with higher adherence, while poor information is associated with lower adherence (Kripalani et al. 2007; Matthes and Albus 2014). Increased adherence, in turn, can help to save costs and to improve patient-relevant outcomes (Sokol et al. 2005; Kripalani et al. 2007; Haynes et al. 2008; Matthes and Albus 2014). Non-adherence, on the other hand, was associated with frequent visits to the doctor and longer treatment times, in addition to a decrease in the effectiveness of treatments and more frequent hospitalizations (Simpson et al. 2006; Howard et al. 2007; Cramer et al. 2007). Recall of drug information correlates with patient participation and patients like to be involved (Dillon 2012; Altin and Stock 2016; Milky and Thomas 2020). Taken together, the prescription talk is a crucial part of physician–patient interaction that helps to ensure safe and effective drug therapy. However, the quality, content, and structure of conversations about newly prescribed medicines vary widely and often the given information is insufficient (Tarn et al. 2006). Physicians are legally obligated to inform patients about a therapy, to explain risks, and to obtain consent for treatment (e.g., German Patients’ Rights Act; Professional Code of Conduct of Physicians in Germany). However, structure and content of respective conversations are not defined in more detail.

We have developed a guide that combines patient-relevant drug information based on the Medication Communication Index (MCI) with the basic steps of shared decision-making (SDM) (Loh et al. 2007; Tarn et al. 2009a; Hauser and Matthes 2017). The guide aims at a conversation in which patients receive satisfying information and are activated to participate in therapy decisions through a structured approach. For our study, we chose GP trainees in community-based primary-care practices, as they are less experienced in managing consultations. The aim of our study was to find out whether our conversation guide is applicable in a GP setting. We also wanted to know whether the guide might be able to influence the patient’s and the doctor’s perception of the conversation compared to unguided conversations conducted as usual. Parts of this work have already been published as a conference abstract (Kirsch and Matthes 2021).

## Material and methods

This is a controlled pilot study conducted in a sequential pre-post design. The applicability and possible effect of a self-developed conversation guide for prescription talks was examined by means of questionnaires.

### The conversation guide

Our conversation guide for prescription talks AMPEL (Arzneiverordnungsgespräche unter Berücksichtigung Medikamentöser Aspekte und der Partizipativen Entscheidungsfindung—ein Leitfaden; English name, Aspects of Medication and Patient participation – an Easy guideLine) is given as a short version in Table 1 (see table S1 for the extensive version). The guide was previously developed (Hauser et al. 2017; Hauser and Matthes 2017). Together with medical students, the guide had been discussed, adjusted, and then tested in simulated prescription talks. In these simulations, the guide proved to be reliable and discriminative when used as a checklist (Hauser et al. 2017). The guide is based on informative aspects of an effective and safe drug therapy, as well as shared decision-making (Charles et al. 1999; Loh et al. 2007; Tarn et al. 2009a).

**Table 1:** Conversation guide for a prescription talk (AMPEL: Aspects of Medication and Patient participation—an Easy guideLine)—short version

Conveying the aim of the conversation (To what extent does the patient want to be involved?)
Underscore communality (Decision should be made or at least supported by both, patient and physician)
Exploration of the patient’s background (e.g., previous knowledge, expectations, circumstances that might affect medication adherence)
Information about treatment options (Goals–duration – names of drugs or drug classes–chances – risks)
Asking for preferences (Asking patient about putative preferences regarding the introduced treatment options)
Negotiation of preferable treatment option(s) (Weighing up pros and cons of the treatment options)
Making a treatment decision (Bringing about a decision, recapitulating the result)
Stipulation about the course of action (e.g., taking instructions, suggesting an evaluation of the decision)

### Inclusion criteria

We recruited GP trainees (i.e., physicians in postgraduate training to become general practitioners) in community-based primary care practices in Cologne. GP trainees were selected because they have less experience in conducting consultations. Thus, on the one hand, they may be more receptive to support. On the other hand, they are more likely not to be biased by an approach they have acquired mainly through routine in their daily practice. Patients were eligible if the physician–patient interaction was about a new drug prescription or a change in drug therapy. The patients had to be full age and cognitively able to make their own decisions regarding a drug therapy. The initial inclusion criterion was being prescribed a long-term therapy, but was expanded to the prescription of new medications for non-chronic conditions as well due to slow recruitment of patients. The participating physicians decided on the eligibility of the patients, informed them about the study, and obtained their consent to participate.

### Study design and procedure

This was a controlled pilot study conducted in a sequential pre-post design. Thus, GP trainees were allowed to participate in both study phases. One main reason for this was to reduce a bias due to interpersonal differences between doctors participating in either the pre- or the post-intervention phase. We did not assign doctors from different practices to either the pre- or the post-intervention group for the same reason and to avoid bias due to differences, e.g., in the respective population of patients. Participating physicians initially conducted prescription talks without knowledge of the guide (pre-intervention phase). They did not know the aims of the study and were only informed that they and their patients would be asked about their “satisfaction with the physician–patient interaction” and that they would receive written recommendations on how to structure prescription talks in the second study phase (post-intervention phase). The intervention phase started after about 6 months with the introduction of the conversation guide to the respective physician and a briefing on how to use it. Within 5 min, the main points of the guide (i.e., relevant drug information and the elements of shared decision-making) were mentioned, but there was no explanation of the theoretical background or the development of the guide. The physicians were asked to read the guide carefully before starting the post-intervention phase and to conduct the subsequent physician–patient conversations in consideration of the guide. Note that the doctors were not aware of the questionnaires that the patients were asked to fill in, nor of the issues raised in them.

### Recruitment of study participants

GP trainees in non-university, community-based primary care practices in Cologne were invited via mail and by phone to participate in the study. Six physicians from five practices were ultimately recruited; four of the physicians participated in both study phases and two of them only in the intervention phase (Table 2). The doctors participated voluntarily and without incentive. They were involved neither in the planning of the study nor in the analysis of the data. The participating doctors selected the patients who met the inclusion criteria, asked them to participate in the study, and, if interested, referred them to an envelope containing written information and the questionnaire. In the patient information, it was pointed out that the aim of the study was to investigate “whether the communication between doctors and patients can be further improved.” Therefore, following the interview to be conducted, “we would like to know how satisfied you are at the moment with the information you have received from your doctor.” The authors of the study were named as contact persons for any queries. The patients handed in the completed questionnaire in an envelope sealed by them, knowing that the doctors had no access to it. Note that the doctors did know neither the written information nor the questionnaire handed over to the patients. Depending on the point in time the patients visited the physician, they were part of the pre- or the post-intervention group, respectively. In the pre-intervention phase, five patients did not complete or did not hand in their questionnaire. For every available patient questionnaire, the corresponding physician questionnaire was available, too.

**Table 2:** Description of conversations and of patients in the pre- and post-intervention phase, i.e., before and after introducing the guide to physicians (absolute numbers and proportions (%) in brackets)

Sample characteristics	Pre-intervention	Post-intervention
Gender		
Female Male	29 (71%) 12 (29%)	17 (74%) 6 (26%)
Average age (years) (range)	44 (20–80)	49 (22–73)
Female Male	42 51	47 55
Prior medication		
Yes No	24 (59%) 17 (42%)	13 (57%) 10 (44%)
Numbers of conversations per physicians A–F		
A B C D E F	11 13 7 10 0 0	4 5 3 6 2 3
Treatment occasion		
Chronic Non-chronic	28 (68%) 12 (29%)	10 (44%) 13 (56%)

### Questionnaires

Immediately after each conversation, patients and physicians each completed a paper questionnaire referring to this talk. The SIMS-D (Satisfaction with Information about Medicine Scale, German version) is a patient questionnaire assessing the extent to which patients feel that they received enough information about a newly prescribed medication. It is comprised of the two subscales, “satisfaction with information about the action and usage of medication” and “satisfaction with information about potential problems of medication,” and the sum of both scales, “satisfaction with information about medicine overall.” With a retest reliability of r > 0.7 and an internal consistency of Cronbach’s α = 0.92, the German version can be considered reliable (Mahler et al. 2009b). Although the SIMS has proven to be valid (Horne et al. 2001), the obtained values have to be interpreted by relating to other inventories or by analyzing differences between groups. The values obtained in our pre-intervention group fit pretty well the data obtained in a non-interventional study on the psychometric properties of the SIMS-D (Mahler et al. 2009b). The KPF-A (Kölner Patientenfragebogen- ambulant) is a survey measuring the satisfaction of patients with various quality dimensions of outpatient care. Seven scales were selected a priori (Table 3). The internal consistency of the scales were reported to be high with Cronbach’s α = 0.87–0.95 (Brinkmann et al. 2007). Similar to the SIMS-D, we used the KPF-A for comparison within our study population.

**Table 3:** Patient satisfaction with quality dimensions of outpatient care (KPF-A) and with information about a prescribed medication (SIMS-D). ^#^Scaling inverted for comparability. *Statistically significant difference when performing Mann–Whitney U test (p < 0.05)

	Pre-intervention phase	Post-intervention phase
KPF-A questionnaire		
Trust in physician (range: 1–4)		
Mean ± standard deviation Mean rank	3.5 ± 0.7 30.4	3.5 ± 0.9 35.0
Neglect by the physician (range: 1–4)^#^		
Mean ± standard deviation Mean rank	3.3 ± 0.7 28.3	3.7 ± 0.5 36.9
Professional competence of the physician (range: 1–4)*		
Mean ± standard deviation Mean rank	3.5 ± 0.5 28.8	3.7 ± 0.6 39.1
Support by the physician (range: 1–4)*		
Mean ± standard deviation Mean rank	3.4 ± 0.7 29.3	3.7 ± 0.7 38.1
Patient activation by the physician (range: 1–4)*		
Mean ± standard deviation Mean rank	2.9 ± 0.8 26.6	3.6 ± 0.8 43.0
Medical information needs (range: 1–2)*		
Mean ± standard deviation Mean rank	1.6 ± 0.4 27.6	1.9 ± 0.2 41.2
Satisfaction overall (range: 1–5)*		
Mean ± standard deviation Mean rank	4.5 ± 0.7 27.0	5.0 ± 0.3 40.8
SIMS-D		
Satisfaction with information about the action and usage of medication (range: 0–9)*		
Mean Minimum Maximum	8 2 9	9 0 9
Satisfaction with information about potential problems of medication (range: 0–8)*		
Median Minimum Maximum	3 0 8	8 0 8
Satisfaction overall (range: 0–17)*		
Median Minimum Maximum	10 4 17	17 2 17

The doctors were asked to fill in a questionnaire for each conversation they had during the study. This questionnaire was designed for the study and contained five items assessing the physician’s satisfaction with the respective prescription talk overall, the conversation technique, the duration of the talk, the outcome of the conversation, and whether the physician was able to clarify everything he or she had set out to do. For the post-intervention phase, nine items on the use of the guide were added. The physicians were asked to state whether they found the guide feasible and helpful, to what extent they followed the guide (overall, regarding the drug-related aspects, regarding the patient participation, regarding the sequence of aspects), whether the duration of a guide-based conversation was perceived longer than usual, and to what extent they considered the procedure and the information to be suitable and relevant for the patient. The items were scored on a verbalized four-point Likert scale (“strongly agree or totally”, “rather agree or mostly”, “rather disagree or somewhat”, “strongly disagree or not at all”). The comprehensibility and unambiguity of the items were confirmed in advance by means of “think-aloud interviews” with ten physicians and fifth-year medical students.

### Data processing

Patient and physician questionnaires of the same conversation were assigned with a pseudonym to be able to match them. Data were obtained and analyzed on the patient level not on medication level. In the SIMS-D, patient responses “too much”, “too little”, or “none received” were coded 0, while “about right” or “none needed” were coded 1. Summing items 1–9 represented the satisfaction with “action and usage of medication” (scores ranging from 0–9; subscale 1). Items 10–17 identified the satisfaction with “potential problems of medication” (scores ranging from 0 to 8; subscale 2). In addition, a total score of all items was calculated for overall satisfaction with information received (Mahler et al. 2009b). According to the KPF manual, the responses to the KPF-A items were coded 1–2, 1–4, or 1–5, respectively (Pfaff et al. 2004). The data was entered by someone not involved in the analysis. The analysis began when all the data was available. Patient-related data was analyzed under pseudonyms. Data was managed and analyzed using Microsoft® Excel 2016, and statistical analyses were performed using SPSS® Statistics for Windows, Version 27.0 (IBM®). The original sheets were archived in paper form. Missing values in the data set led to exclusion of the affected scale from the analysis.

### Statistical analysis and ethics

As a pilot study, the investigation aimed at feasibility and acceptance. Furthermore, the satisfaction of patients and doctors was exploratively surveyed in order to test the kind and extent of effects that may be expected. Thus, there was no sample-size estimation. Due to non-normal data distribution according to Kolmogorov–Smirnov, the non-parametric Mann–Whitney U test for independent samples was applied. Statistical significance was assumed for p values < 0.05. Comparison of groups was performed for the entire study sample and for chronic and non-chronic treatment occasions separately. Effect sizes as Cohen’s d were calculated from Mann–Whitney U tests. The local ethics committee did not raise any concern against the study (ref.: 17–290).

## Results

### Conversations and patient characteristics

In total, 128 questionnaires from 64 physician–patient conversations were collected (Table 2). From the pre-intervention phase, patient and physician questionnaires on 41 conversations were available, and from the post-intervention phase, i.e., after the guide had been introduced, there were data on 23 conversations available. There was no difference in gender (71% and 74% female) or age (44 ± 17 and 49 ± 17 years) between patients in the two study phases. Fifty-nine percent from the pre- and 57% from the post-intervention phase were already taking medication regularly before the prescription talk. Long-term therapy was prescribed in 72% and 44% of the cases, respectively. In 59% of all conversations, there was a chronic reason for treatment and in 39% a non-chronic reason for treatment (information for one patient was missing).

### The conversations from the patients’ perspective

**Fig. 1:**
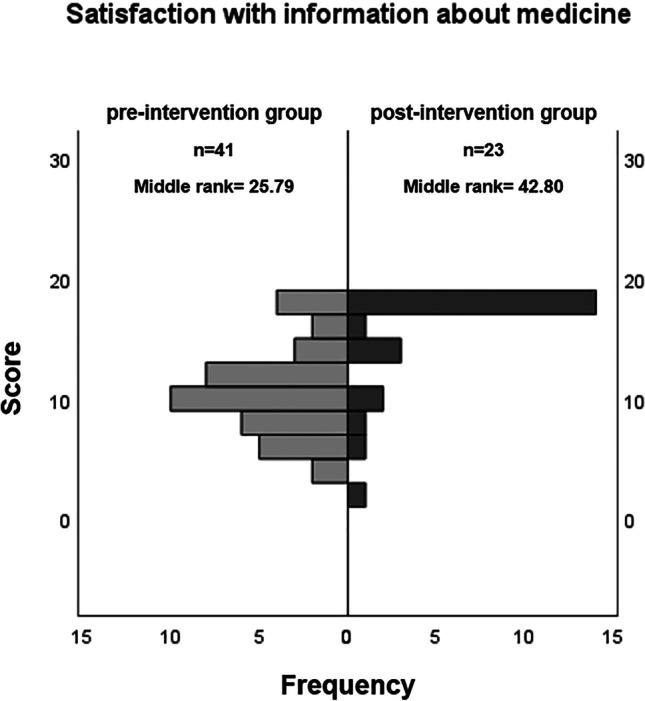
Patient satisfaction with information about their medication. Frequency distribution of the overall assessment of the medication information received during the prescription talk, as surveyed with the SIMS-D questionnaire. The physicians conducted the conversations without (pre-intervention group) or with knowledge of the guide (post-intervention group). The difference between the two groups was statistically significant (p < 0.05 in the Mann–Whitney U test)

**Fig. 2:**
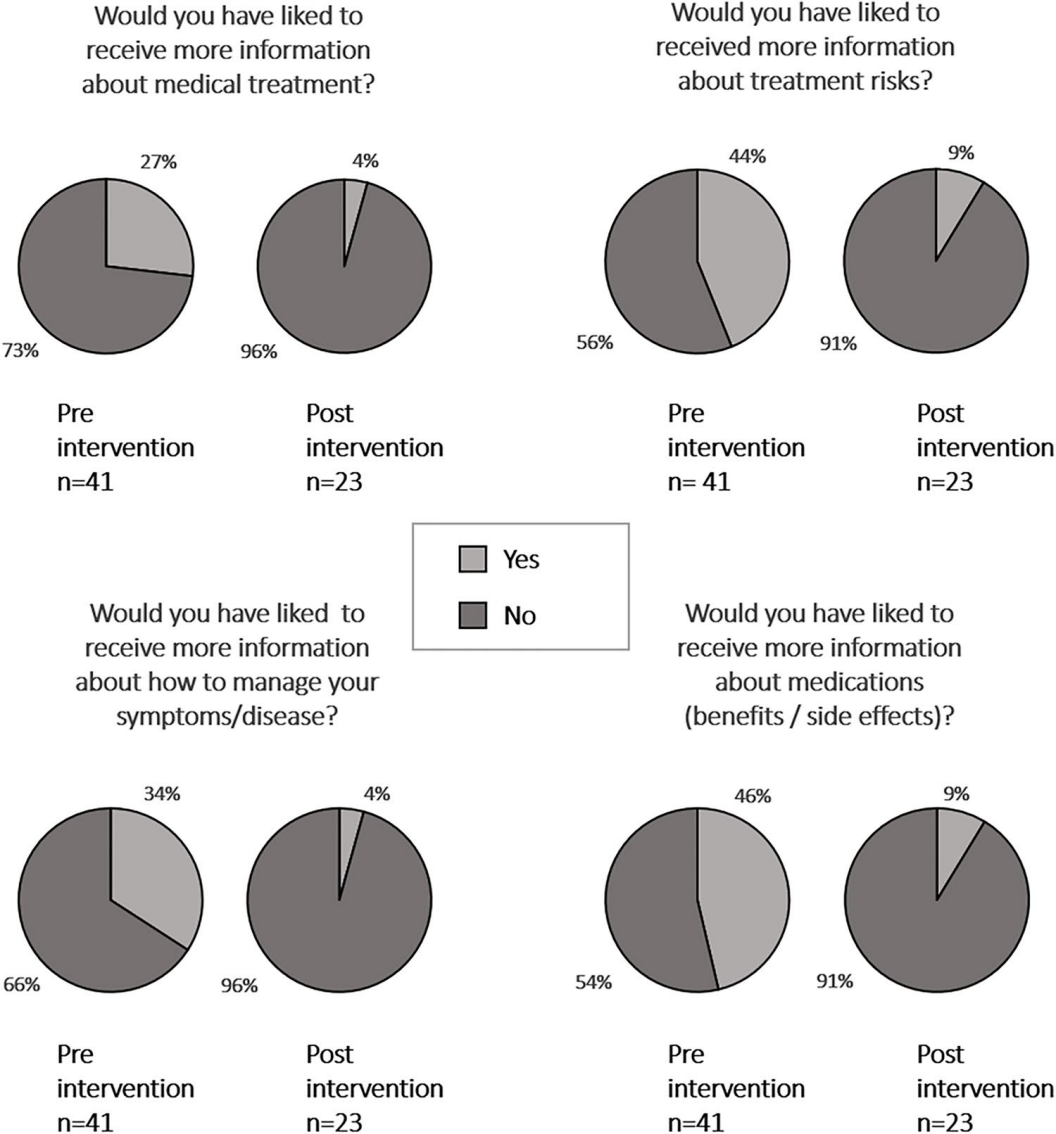
Patients’ medical information needs after the prescription talk. Items of the “medical information needs” scale of the KPF-A questionnaire are displayed. The physicians conducted the conversations without (pre-intervention) or with knowledge of the guide (post-intervention). The difference of the sum scores between pre- (n = 41) and post-intervention group (n = 23) was statistically significant (p < 0.05 in the Mann–Whitney U test)

**Fig. 3:**
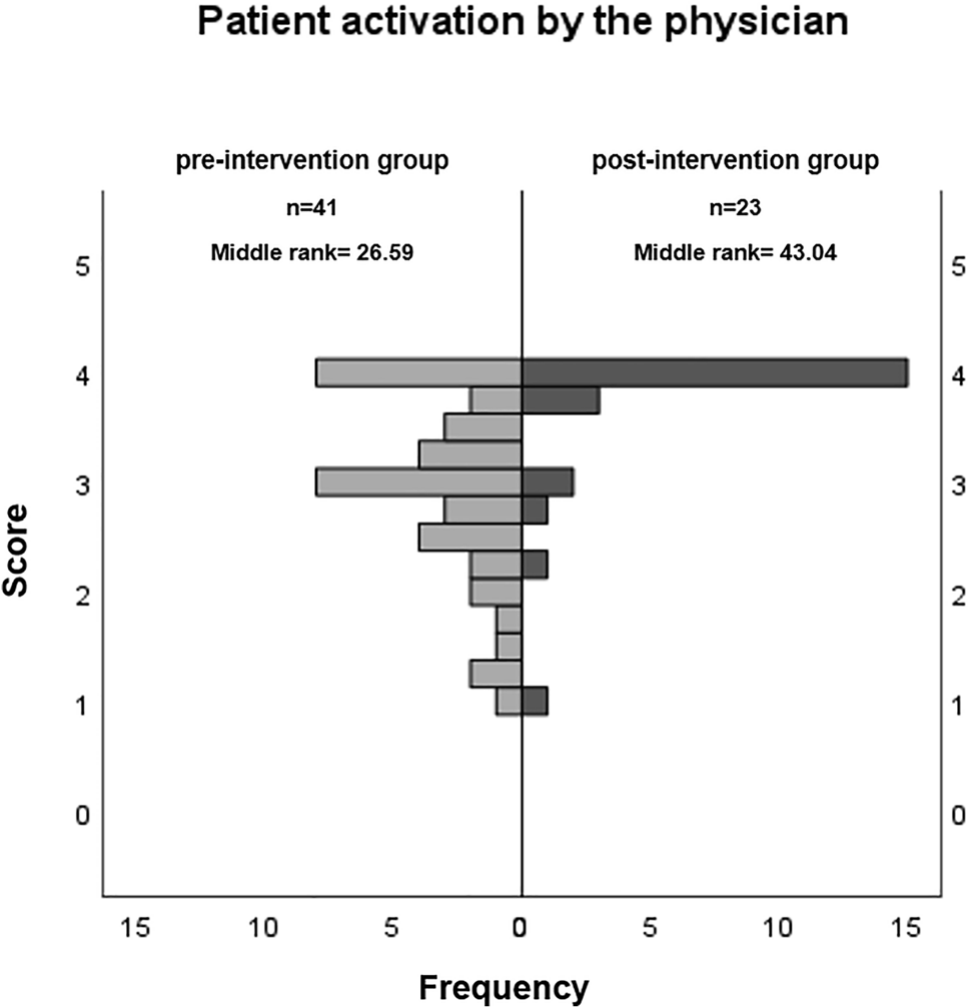
Patient activation by the physician in the prescription talk. Frequency distribution of the mean score of the scale “patient activation by physicians” of the KPF-A questionnaire. The physicians conducted the conversations without (pre-intervention group) or with knowledge of the guide (post-intervention group). Patients in the post-intervention group evaluated their activation by physicians significantly higher (p < 0.05 in the Mann–Whitney U test)

Satisfaction with medication information (SIMS-D) was higher in the post-intervention phase than in the pre-intervention phase. This difference was statistically significant for the two subscales “effect and use” (effect size d = 0.61) and “possible problems” (d = 0.93) (Fig. 1) as well as for overall satisfaction (d = 1.0) (Table 3). The proportion of patients who reported an unmet need for information after the prescription talk (KPF-A) decreased significantly (Fig. 2). According to the KPF-A, overall satisfaction with the conversation was significantly higher during the post-intervention phase than in the pre-intervention phase (d = 0.78) (Table 3). With regard to the elements of shared decision-making that our guide included, the subscales “support by the physician” and “patient activation by the physician” were of particular interest. Both subscales showed a statistically significant increase in the intervention phase (d = 0.47 and 0.94, respectively) (Fig. 3; Table 3). Ratings of “professional competence” were higher, too (d = 0.55). Separate analyses of conversations on chronic and non-chronic treatment occasions showed similar effects. Despite smaller numbers of cases, some of these differences reached statistical significance, too (chronic, all SIMS-D scales as well as the KPF-A scales “patient activation,” “support,” “professional competence”; non-chronic, KPF-A scales “information needs,” “overall satisfaction”).

### The conversations from the physicians’ perspective

**Fig. 4:**
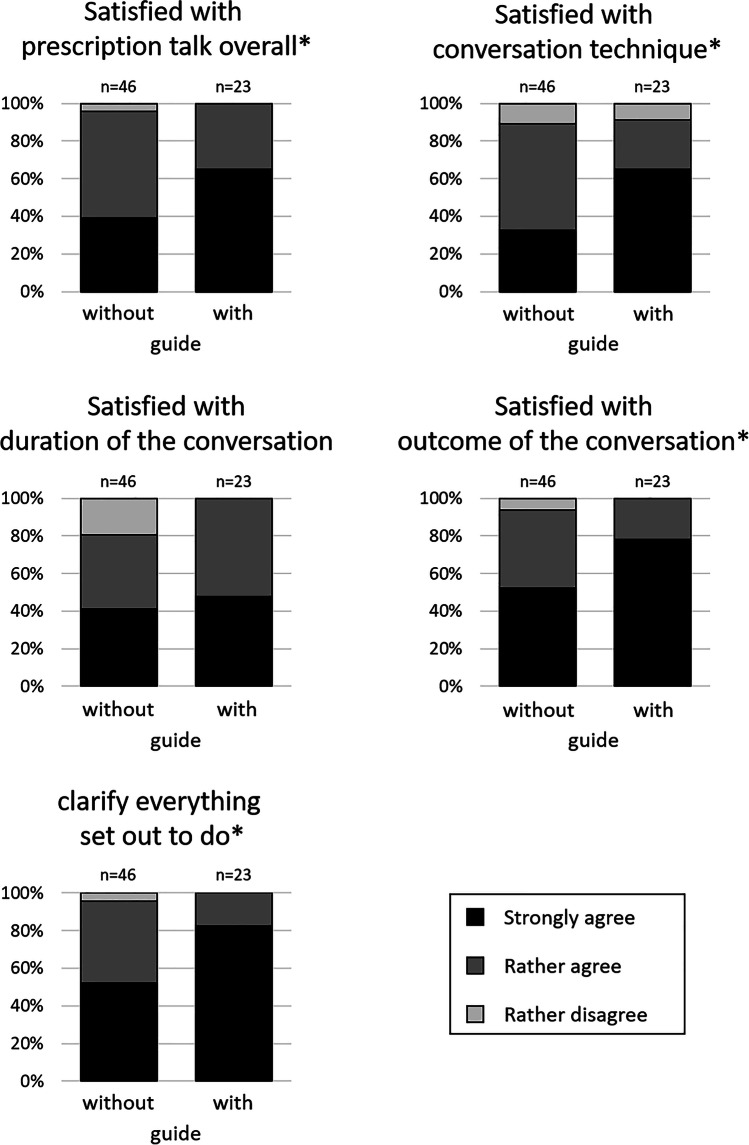
Physicians’ evaluation of the prescription talks. The physicians conducted the conversations without (pre-intervention phase, n = 46) or with knowledge of the guide (post-intervention phase, n = 23) and rated five statements on satisfaction with the conversation using a verbalized four-point Likert scale. The percentage of times each option was selected is shown (“does not apply at all” was never selected). The differences between pre- and post-intervention phase were statistically significant (* p < 0.05 in the Mann–Whitney U test), excluding the satisfaction with the duration of the conversation

In the intervention group, physicians’ satisfaction with the prescription talk overall (d = 0.46), with the way they conducted the conversation (d = 0.51), and with the outcome of the conversation (d = 0.46) was significantly higher (Fig. 4). Furthermore, after guide-based conversations, physicians more often felt that they had been able to clarify everything they had set out to do (d = 0.52). Satisfaction with the duration of the conversation was high overall with no statistically significant difference between pre- and post-intervention phase. None of the conversations was rated as “too long” and only one in the pre-intervention phase was rated as “too short”.

In the vast majority of interviews, physicians “completely” or “rather” agreed that the guide was feasible (21 out of 23) and helpful (22 out of 23), respectively. According to their own statements, physicians used the guide in 22 of the 23 conversations. The physicians followed the guide even more closely for aspects concerning patient participation (23 out of 23) than for aspects concerning information on medication (19 out of 23). Regarding the question whether the various aspects were addressed in the order suggested by the guide, physicians stated two times “completely” and five times “mostly,” while in 14 cases, the response was “mostly not,” and in two cases “not at all.” With respect to the statement that the procedure the guide provided was suitable for the patient, the physicians agreed “completely” after ten conversations and “mostly” in twelve cases. After 14 conversations, doctors stated that the provided information was “completely” relevant for the patients, and in another five cases, they rated it as “mostly relevant.” Regarding the statement that a conversation had become longer due to the guide, physicians “mostly” agreed in ten cases, and in one the physician agreed “completely.”

## Discussion

*On the ward of a geriatric clinic, the pharmacist talks to an 83-year-old patient about her medication. When asked about pantoprazole, the patient reports that she does not take it at all. She had read on the internet that the drug caused osteoporosis. In the patient’s file, the pharmacist reads that the patient has type B gastritis.*

This short real-life example shows how complex safe drug therapy can be. The conversation with the pharmacist reveals the patient’s non-adherence. She has informed herself on the internet. A look at the file questions the indication for the medication, because other components of an eradication therapy were missing. On the other hand, it cannot be ruled out that the patient mistakenly continued to take the proton pump inhibitor beyond the end of the eradication therapy. Perhaps, a better prescription talk would have helped. In an effective prescription talk, a patient would be informed about the reason for and the course of a treatment as well as possible risks in order to decide on a therapy that also meets the patient’s individual needs and concerns. At the end of a shared decision-making process, there would then be an appointment to re-evaluate the therapy in order to address any changes or new concerns that may have arisen on the part of the patient. The conversation guide we have developed aims to help improve prescription talks by keeping patients well informed and actively involved.

In this pilot study, patients were more satisfied with the doctor-patient communication when prescription talks were conducted using our guide AMPEL. They felt better informed and more actively involved in the conversation. In most cases, the physicians used the guide and rated it as feasible and helpful.

Regarding drug information, our guide is based on the Medication Communication Index (MCI) developed by Tarn et al. (2009a). The authors found higher MCI scores to be associated with better patient ratings of information about new prescriptions (Tarn et al. 2013). While patient satisfaction with general information in doctor-patient contacts is high overall, patients would like to know more about possible adverse effects and drug-related problems (Mahler et al. 2009a). Although patients consider this information as particularly important (Ziegler et al. 2001; Twigg et al. 2016; Kusch et al. 2018), it is often missing in prescription talks (Tarn et al. 2006; Richard et al. 2017). The fear that informing patients about possible adverse effects of a treatment could have a negative impact on medication adherence, occurrence of suspected side effects and clinical outcomes, could not be confirmed (Jose and AlHajri 2018). In our study, particularly, the satisfaction with the information about potential problems increased significantly when the conversation was based on the guide. Compared to the drug-related problems, satisfaction with the use-related information was higher in the pre-intervention phase, but it also improved with the guide.

In our guide, drug information is linked to the essential steps of shared decision-making. Shared decision-making aims at a joint decision between the doctor and the patient (e.g., about a therapy) and is considered a gold standard of doctor-patient communication (Elwyn et al. 2012). In fact, patients want to be involved in medical decisions that affect them and are more satisfied when they are involved in these decisions (Altin and Stock 2016; Milky and Thomas 2020). Our survey showed that patients felt more activated and better supported during a guide-based conversation. Patient participation increases the amount of information a patient remembers and is associated with patient-relevant outcomes (Parchman et al. 2010; Hauser et al. 2015; Richard et al. 2017). For information-based decision-making, or at least informed consent, active patient participation is particularly important (Richard et al. 2017). In the present study, only the level of (perceived) patient activation was surveyed. However, it has been shown that a physician communication style promoting shared decision-making is associated with increased patient activation, which in turn is directly linked to enhanced therapy adherence (Parchman et al. 2010).

During the post-intervention phase, the physicians mostly considered the guide and rated it as feasible and helpful. They followed the guide with regard to patient participation even more than with regard to relevant drug-related information. Perhaps, drug information has already been considered more during the pre-intervention phase, possibly against the legal background, among other things. Patient participation in terms of shared decision-making may have been rather new or unfamiliar and therefore drew more physicians’ attention to these aspects of the guide. Note that the physicians participating in our study have been GP trainees with limited practical experience. Although patient-centeredness is hampered by physicians’ fear of an increased time burden, numerous studies have failed to support this concern (Légaré and Witteman 2013). As well, deepening the discussion of drug-related aspects seems to prolong physician–patient contacts only slightly overall. In physician–patient contacts that lasted a mean of 15.9 min, an average of only 49 s was spent discussing a new medication. Even when all aspects of the MCI were covered, the average duration was just 85 s (Tarn et al. 2008). In our study, for almost half of the conversations, physicians stated that it took longer due to the use of the guide. However, there was no difference between the pre- and the post-intervention phases in terms of physicians’ satisfaction with the duration of the talks.

It is not really surprising that many interviews deviated from the order of aspects as given in the guide. Physician–patient communication must be dynamic and flexible. That our guide can live up to this claim is shown by the fact that, overall, the doctors in our study largely used it. Regardless of the sequence of items, they found it mostly suitable and relevant for their patients.

Compared with more complex interventions (e.g., training of physicians), the present study suggests a surprisingly high effectiveness of this simple intervention (Ledford et al. 2014). Although addressing another outcome, studies on interventions aiming to increase medication adherence have already shown that simple, low-effort interventions can be just as effective or even more effective than complex approaches (Kripalani et al. 2007; Matthes and Albus 2014).

## Limitations

There are some limitations of our study to be considered. Physicians were asked to read the guide carefully, but it was not checked to what extent they did do so or whether they did so at all. The implementation of the guide was only evaluated by means of self-assessment. We cannot exclude a bias by physicians participating in both the pre- and the post-intervention phase. However, participating residents did know neither the aims of the study nor the questionnaires patients were asked to fill in. They were only told that they and their patients would be asked about their “satisfaction with the physician–patient interaction” and that the physicians would receive written recommendations on how to structure prescription talks in the second study phase. Of note, doctors participated voluntarily, without any incentive, and were not involved in data analysis. We chose GP trainees for our study because they maybe be more receptive for support on the one hand and less biased by daily routine on the other hand. Unlike GPs, trainees lack the long-term relationship to their patients and thus still may have to earn their trust. Communication skills of trainees might have improved independent of our study intervention. Since GP trainees in community-based primary care practices in Cologne are of course not representative of physicians in general, future studies have to explore whether the data obtained can be transferred to other local or medical conditions. Patient information and activation were not measured directly. However, here, a perceived enhancement can already be considered an improvement in patient care (Rummer and Scheibler 2016). We have no data on the adherence of the patients in our study. However, patient information and activation have been associated with an increase in adherence in other studies (Kripalani et al. 2007; Parchman et al. 2010).

## Conclusion

In this study, our conversation guide was applicable in medical practice. Furthermore, its use appeared to be associated with significantly increased satisfaction with physician–patient conversations among both doctors and patients. The promising results justify further studies to verify whether the increase in perceived patient information and activation can be confirmed and validated and to investigate whether it may influence treatment adherence. We invite physicians to try our guide in everyday medical practice.

## Supplementary information

Below is the link to the electronic supplementary material.

Supplementary file1 (DOCX 16 KB)

## Declarations

### Disclaimer

The authors declare that all data were generated in-house and that no paper mill was used.
