# Supplementary material for: Aspects of Medication and Patient participation—an Easy guideLine (AMPEL). A conversation guide increases patients’ and physicians’ satisfaction with prescription talks
Source: Naunyn Schmiedebergs Arch Pharmacol. 2021 Jun 9;394(8):1757–67. doi: 10.1007/s00210-021-02107-0 (PMC8298249; doi:10.1007/s00210-021-02107-0)
Supplement: Supplementary file 1 — Supplementary file1 (DOCX 16 KB) [file 210_2021_2107_MOESM1_ESM.docx]

**Table S2:** Conversation guide for a prescription talk (AMPEL: **A**spects of **M**edication and **P**atient participation – an **E**asy guide**L**ine) – long version (rear side of the handout for doctors)

| **Conveying the aim of the conversation**   - - Tell the patient that one has to come to a treatment decision.   - Ask the patient whether and how far he or she wants to participate in the decision process. |
| --- |
| **Underscore communality**   - - Tell the patient that the decision should be made or at least supported by both, patient and physician. |
| **Exploration of the patient’s background**   - - Ask the patient about   - knowledge and attitude towards his or her disease.   - comprehension, attitude and expectations regarding a therapy.   - problems or circumstances that might affect medication adherence. |
| **Information about treatment options**   - - Explain relevant treatment options to the patient and   - mention or explain purpose or justification of the treatment and / or therapeutic goals.   - tell him or her about duration of therapy (therapies).   - give (generic or trade) names of drugs or drug classes.   - delineate advantages and chances of the treatment options.   - describe risks and adverse effects of the treatment options.   - point out probability and extend of risks and adverse effects as well as of the expected benefits in a comprehensible and demonstrative manner. |
| **Asking for preferences**   - - Ask the patient about putative preferences regarding the introduced treatment options. |
| **Negotiation of the preferable treatment option(s)**   - - Negotiate a treatment option with the patient and thereby   - help him or her with weighing up pros and cons of the treatment options.   - help him or her weighing up how far treatment options might match or not match his or her way of life / lifestyle. |
| **Making a treatment decision**   - - Bring about a decision for one treatment option and   - recapitulate the result / the decision made up together. |
| **Stipulation about the course of action**   - - Bring about a stipulation regarding the realization of the treatment decisions and thereby   - tell the patient about details of drug use (e.g. No. of tablets, timing, dosing interval).   - suggest an evaluation of the decision, e.g. by making a follow-up appointment. |
